# Wilms Tumor-1 (*WT1*) rs16754 Polymorphism and Clinical Outcome in Acute Myeloid Leukemia

**DOI:** 10.4274/tjh.galenos.2018.2018.0277

**Published:** 2019-02-07

**Authors:** Mani Ramzi, Mohamad Moghadam, Nader Cohan

**Affiliations:** 1 Shiraz University of Medical Sciences, Hematology Research Center, Shiraz, Iran

**Keywords:** Polymorphism, Acute myeloid leukemia, Clinical outcome

## To the Editor,

Cytogenetic evaluation and risk stratification are important steps in the classification of acute myeloid leukemia (AML) and also in determining the best treatment modality. Although cytogenetically normal acute myeloid leukemia (CN-AML) represents a large group of adult patients with AML (about 45% of AML patients), several mutations in some genes including *FLT3, NPM1, CEBPA*, and *WT1* are associated with risk assessment and clinical outcome [1,2]. The *WT1* gene is located on chromosome 11p13 and was detected first as a tumor-suppressor gene in Wilm’s tumor; it also has a regulatory role in normal and malignant hematopoiesis [3]. *WT1* gene mutations in CN-AML were reported in about 10% of patients and its role as a sole prognostic factor is controversial [4,5,6]. The most important *WT1* gene mutations associated with AML are clustered in the hotspot region of exon 7 and a synonymous single nucleotide polymorphism (SNP), rs16754, is also located in this region. Some studies were done for evaluation of *WT1* rs16754 polymorphism effects on clinical response in AML patients. Most studies confirmed that rs16754 (A>G) in the hotspot region of exon 7 is a predictive independent positive prognostic factor in AML patients. Two meta-analyses were published for evaluation of these results. Megías-Vericat et al. [7] performed the first meta-analysis in this regards with a total of 3618 included patients from 14 cohort studies. They found that the overall survival (OS) at 5 years was significantly higher in patients with the variant allele (G) together with higher disease-free survival (DFS) with the variant allele. Although they did not find any significant effect of this variant on complete remission (CR), they stated that the lack of an observable effect could have been a result of the small number of studies that evaluated it. Long et al. [8] also published a meta-analysis in this regard, addressing 12 publications with 3903 included patients. Although some of the publications and included patients in those two studies may have been the same, they found the same results for the significant effects of *WT1* rs16754 polymorphism on OS and relapse-free survival. In a newly published study Petiti et al. [9] also showed that patients with mutated genotypes (GG and GA) have a significantly better OS and event-free survival (EFS) compared with patients with wild AA mutation (84 vs. 9 months for OS and 23 vs. 3 months for EFS). They discussed that they used peptide nucleic acid-directed PCR clamping technology for this study. 

In a prospective study, we evaluated the effects of the rs16754 SNP on clinical outcome in CN-AML patients. From December 2012 to December 2016, a total of 108 untreated adult patients who were referred to Nemazee Hospital, Shiraz University of Medical Sciences, and diagnosed with CN-AML were included. The exclusion criterion was abnormality upon karyotyping. The diagnosis was based on peripheral blood and bone marrow aspiration confirmed by immunophenotyping and the treatment was based on standard induction chemotherapy, which consisted of daunorubicin at 45 mg/m^2^ on days 1 to 3 and cytarabine at 100-200 mg/m^2^ on days 1 to 7, followed by high doses of a cytarabine-based consolidation phase (cytarabine at 3 g/m^2^ every 12 h for 3 days, repeated for 3 to 6 cycles). Informed consent was obtained from all patients and the work was carried out in accordance with the code of ethics of the World Medical Association (Declaration of Helsinki) for experiments in humans. The rs16754 (A>G) polymorphism in exon 7 was evaluated in all patients by direct sequencing of the amplified and purified PCR products. OS and DFS were assessed for evaluation of clinical outcome. The allele frequency of the A allele was 181 and that of the G allele was 35. The major genotype (AA) was found in 79 patients and a minor genotype in 29 patients (AG and GG in 23 (21.3%) and 6 (5.5%) patients, respectively). We found no significant difference between patients with the wild and variant allele based on CR and relapse as well as OS and DFS, which may be a result of the small number of included patients. The results are shown in Table 1. Although the exact reason for the effects of this SNP on clinical outcome in AML patients is unclear, different hypotheses exist, including increased rate of translation and protein folding in mutated genotypes, disequilibrium linkage of this SNP with another genetic variant that affects drug metabolism, and greater sensitivity of mutated genotypes to cytarabine [7,10]. In conclusion, the *WT1* rs16754 polymorphism may be a reliable independent prognostic factor in AML and could be assessed for risk stratification in this group of patients, although more studies including larger study groups are needed for more evaluation and discussion.

## Figures and Tables

**Table 1:**
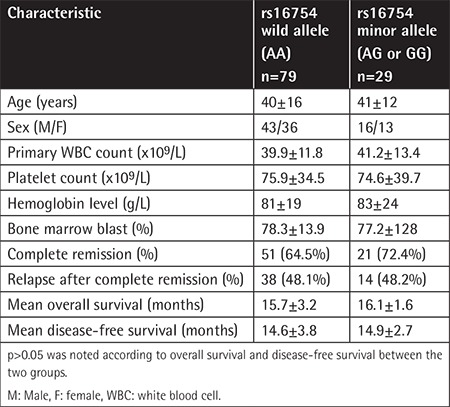
Clinical and laboratory data according to rs16754 wild and minor alleles.
